# Molecular Remodeling of Peritumoral Tissue in Clear Cell Renal Cell Carcinoma: Insights into Inflammaging and Prognostic Markers

**DOI:** 10.3390/cancers18030414

**Published:** 2026-01-28

**Authors:** Giuseppe Stefano Netti, Federica Spadaccino, Giuseppe Lucarelli, Valeria Catalano, Andrea Checchia, Alessandra Stasi, Federica De Luca, Valentina Camporeale, Giorgia Leccese, Roberto Cuttano, Dario Troise, Barbara Infante, Giuseppe Carrieri, Walter J. Storkus, Giovanni Stallone, Elena Ranieri

**Affiliations:** 1 Unit of Clinical Pathology, Department of Medical and Surgical Sciences, University of Foggia—University Hospital “Policlinico Riuniti”, Viale Luigi Pinto, 71122 Foggia, Italy; federica.spadaccino@unifg.it (F.S.); valeria.catalano@unifg.it (V.C.); federica.deluca@unifg.it (F.D.L.); valentina.camporeale@unifg.it (V.C.); giorgia_leccese.555553@unifg.it (G.L.); roberto.cuttano@unifg.it (R.C.); elena.ranieri@unifg.it (E.R.); 2 Center for Research and Innovation in Medicine (CREATE), Department of Medical and Surgical Sciences, University of Foggia—University Hospital “Policlinico Riuniti”, Viale Luigi Pinto, 71122 Foggia, Italy; 3 Unit of Urology, Department of Precision and Regenerative Medicine and Ionian Area (DiMePRe-J), University of Bari “Aldo Moro”, Policlinico, Piazza Giulio Cesare 11, 70124 Bari, Italy; giuseppe.lucarelli@uniba.it; 4 Urology Unit, IRCCS Istituto Tumori “Giovanni Paolo II”, 70124 Bari, Italy; 5 Unit of Urology, Department of Medical and Surgical Sciences, University of Foggia—University Hospital “Policlinico Riuniti”, Viale Luigi Pinto, 71122 Foggia, Italy; andrea_checchia.531401@unifg.it (A.C.); giuseppe.carrieri@unifg.it (G.C.); 6 Unit of Nephrology, Dialysis and Transplantation, Department of Precision and Regenerative Medicine and Ionian Area (DiMePRe-J), University of Bari “Aldo Moro”, Policlinico, Piazza Giulio Cesare 11, 70124 Bari, Italy; alessandra.stasi@uniba.it; 7 Unit of Nephrology, Dialysis and Transplantation, Advanced Research Center on Kidney Aging (A.R.K.A.), Department of Medical and Surgical Sciences, University of Foggia—University Hospital “Policlinico Riuniti”, Viale Luigi Pinto, 71122 Foggia, Italy; dario.troise@unifg.it (D.T.); barbara.infante@unifg.it (B.I.); giovanni.stallone@unifg.it (G.S.); 8 Dermatology, Immunology, Pathology and Bioengineering, University of Pittsburgh Medical Center (UPMC) Hillman Cancer Center, University of Pittsburgh School of Medicine, Pittsburgh, PA 15232, USA; storkuswj@upmc.edu

**Keywords:** clear cell renal cell carcinoma (ccRCC), peritumoral tissue, pentraxin 3 (PTX3), inflammaging, senescence-associated secretory phenotype (SASP), interleukin 6 (IL-6), p21/CIP1/WAF1, p16/INK4a

## Abstract

**Background/Objectives**: Renal cell carcinoma (RCC) is a common and often asymptomatic malignancy with limited treatment options for advanced stages. Chronic inflammation and cellular senescence—collectively termed “inflammaging”—are emerging as key contributors to tumor progression. This study aimed to investigate the expression of inflammaging-related markers in RCC tissues, focusing on the role of PTX3, IL-6, and senescence-associated proteins in the tumor microenvironment. **Methods**: A retrospective cohort of 57 patients with clear cell RCC who underwent nephrectomy was analyzed. Formalin-fixed paraffin-embedded samples from tumor, peritumoral, and normal renal tissues were examined using confocal immunofluorescence microscopy to assess PTX3, IL-6, p21, and p16 expression. Senescence-associated β-galactosidase staining was performed to identify senescent cells. Serum IL-6 levels were measured by ELISA, and survival analysis was conducted using Kaplan–Meier curves and Cox regression analysis. **Results**: PTX3 and IL-6 were significantly upregulated in both peritumoral and tumor tissues compared to normal kidney samples (*p* < 0.001). Expression of senescence markers p21 and p16 were elevated in peritumoral areas (*p* < 0.001) as compared to normal renal tissues, but their expression was reduced or absent in the tumor core. High-grade and high-stage tumors exhibited stronger PTX3 and IL-6 expression and lower levels of cell cycle inhibitors (*p* < 0.001). Patients with elevated serum IL-6 levels had significantly lower 5-year cancer-specific survival (*p* < 0.005) and shorter progression-free survival (*p* < 0.001). **Conclusions**: Our findings suggest that peritumoral tissue in RCC exhibits a senescent and proinflammatory phenotype that may support tumor progression. PTX3 and IL-6 are potential biomarkers of disease severity and prognosis. Targeting inflammaging pathways could offer new therapeutic strategies for RCC, particularly in aggressive disease forms.

## Simple Summary

Renal cell carcinoma is a common and often silent form of kidney cancer. This study explores how chronic inflammation and cellular aging—known together as “inflammaging”—may influence the development and progression of this disease. By analyzing tissue samples from patients, specific molecules linked to inflammation and cell aging have been shown to be more active in structurally normal regions surrounding the tumor. These findings suggest that peri-tumoral tissue may contribute to cancer growth. Understanding this process could lead to improvements in the detection and treatment of kidney cancer, especially in more aggressive cases.

## 1. Introduction

Renal cell carcinoma (RCC) is the most common form of kidney cancer, accounting for approximately 3–4% of adult malignancies in Western countries [1,2,3]. RCC incidence is higher in males and continues to rise annually by 2–4%, with peak mortality observed in individuals aged 55–65 years [4,5]. RCC often presents asymptomatically, with only 10% of patients exhibiting the classic triad of hematuria, flank pain, and palpable mass. Consequently, diagnosis is frequently incidental during imaging for unrelated conditions [5].

Risk factors for RCC include advanced age, obesity, hypertension, smoking, and chronic kidney disease, although hereditary forms also exist [6]. RCC is notably resistant to chemotherapy and radiotherapy, making surgical intervention—radical or partial nephrectomy—the primary treatment option [7]. While surgery achieves 5-year survival rates of 70–80% in localized disease, metastatic renal cell carcinoma remains associated with poorer outcomes [7]; nonetheless, immune checkpoint inhibitor–based combinations have substantially improved long-term survival, with 5-year overall survival rates approaching 30–40% in selected patient populations. The different prognosis between localized and metastatic RCC underscores the need for novel biomarkers and diagnostic strategies to improve early RCC detection and its therapeutic targeting. Advances in understanding RCC pathogenesis have led to the development of targeted therapies, including anti-angiogenic agents, mTOR inhibitors, and immune checkpoint inhibitors [8].

The tumor microenvironment (TME) in RCC is a dynamic and heterogeneous network of cellular and extracellular components. Tumor cells influence the TME directly through local cytokine and growth factor secretion, and indirectly by inducing hypoxia and recruiting immune cells [9]. While acute inflammation may suppress tumor development, chronic inflammation paradoxically promotes tumor progression by fostering a pro-inflammatory milieu rich in cytokines and growth factors [10,11].

PTX3 is a long pentraxin involved in innate immunity and inflammation. Unlike short pentraxins such as CRP, immune and epithelial cells produce PTX3 locally, including renal tubular cells, in response to inflammatory stimuli [12,13,14,15]. PTX3 plays a dual role in cancer biology, acting as either a tumor suppressor or promoter depending on system context [16,17]. It influences tumor progression through modulation of the PI3K/AKT/mTOR pathway and contributes to processes such as epithelial-to-mesenchymal transition (EMT), angiogenesis, and immune evasion [18,19,20,21,22,23].

In genitourinary cancers, PTX3 has emerged as a potential biomarker of poor prognosis. Its expression correlates with disease severity and outcomes in patients with prostate or renal cancer [24,25,26,27,28,29,30,31,32,33,34,35]. In RCC, PTX3 is upregulated in tumor tissues and serum, particularly in high-grade tumors, where it is associated with complement system activation and inflammatory signaling [35].

Senescence is a state of permanent cell cycle arrest triggered by stressors such as DNA damage, oxidative stress, and oncogene activation [36,37,38,39,40,41]. Senescent cells remain metabolically active and secrete a range of pro-inflammatory factors known as the senescence-associated secretory phenotype (SASP), including IL-6, IL-8, and matrix metalloproteinases [42,43,44,45,46,47]. These factors can reinforce senescence in neighboring cells and contribute to tumor progression and therapy resistance [48,49,50,51].

In RCC, senescence and SASP are increasingly recognized as contributors to the inflammatory TME. Markers such as p21 and p16 are involved in regulating cell cycle arrest and are differentially expressed in peritumoral versus tumor core tissues, suggesting a spatial modulation of senescence within the tumor landscape [52,53,54,55,56,57,58].

This study aimed to investigate the expression of inflammaging-related markers in renal cell carcinoma tissues, focusing on the role of PTX3, IL-6, and senescence-associated proteins in the tumor microenvironment and peritumoral regions. By characterizing their spatial distribution and correlation with tumor grade and patient outcomes, the study investigates the hypothesis that chronic inflammation and cellular senescence affect ccRCC disease progression and prognosis.

## 2. Materials and Methods

### 2.1. Study Population and Sample Collection

Among all patients undergoing radical or partial nephrectomy for renal masses at the Urology Unit of University Hospital “Ospedali Riuniti” of Foggia (Italy) between January 2016 and June 2020, this retrospective, single-center, observational cohort study included 57 consecutive patients who met the inclusion criteria reported below and provided signed informed consent to participate in the present study. Briefly, all enrolled patients had histologically confirmed ccRCC and were older than 18 years at time of surgery. For each enrolled patient, both FFPE normal and cancer renal tissues and serum samples were available for analyses. Patients with severe health conditions at the time of hospital admission and a follow-up of less than 6 months were excluded from the study. Detailed clinical and pathological characteristics of the patients are summarized in Table 1. Normal renal tissue used as baseline reference was obtained from 10 age- and sex-matched individuals undergoing renal biopsy at Nephrology Unit of University Hospital “Ospedali Riuniti” of Foggia for suspected chronic nephropathy but showing normal histology, as assessed by two independent nephro-pathologists. In the current study, peritumoral tissue was defined as renal parenchyma located 1–2 mm from the tumor margin and histologically confirmed to be free of neoplastic infiltration. Tumor tissue consisted of histologically confirmed ccRCC samples. Two independent pathologists confirmed the presence of ccRCC in the neoplastic tissues and excluded tumor cell infiltration in the healthy specimens by H&E staining. Tumor and peri-tumoral tissues were collected, and stored frozen at −80 °C according to standardized procedures.

In addition, serum samples were collected with consent from 57 patients who underwent radical or partial nephrectomy for ccRCC at Urology Unit of University Hospital “Policlinico” of Bari. For serum IL-6 comparisons, samples from 40 volunteers without evidence of malignancy served as controls. Serum samples were obtained from each patient at the time of nephrectomy and stored at −30 °C.

All RCC patients were preoperatively staged by thoraco-abdominal Computed Tomography or Magnetic Resonance Imaging. Tumor staging was reassigned according to the seventh edition of the AJCC-UICC TNM classification. The 2016 World Health Organization and Fuhrman classifications were used to attribute histological type and nuclear grade, respectively.

The present study involving human participants was approved by the local ethical committee (Decision n. 152/CE/2014 of 3 September 2014; Ethical Committee at the University Hospital “Ospedali Riuniti” of Foggia). All procedures performed were in accordance with the ethical standards of the Declaration of Helsinki and all the enrolled patients provided their informed written consent to participate in this study.

### 2.2. Senescence-Associated SA-β-Gal Staining

Senescence-associated SA-β-Gal staining was performed using a commercial kit per the manufacturer’s protocol (Cell Signalling Technology, Danvers, MA, USA), as described [59]. After washing with PBS (pH 6.0), frozen tissue sections were fixed in 4% paraformaldehyde for 15 min and stained with freshly prepared SA-β-Gal solution (1 mg/mL X-gal, 40 mM citric acid/sodium phosphate (pH 6.0), 5 mM potassium ferrocyanide, 5 mM potassium ferricyanide, 150 mM NaCl, and 2 mM MgCl) at 37 °C for 12–16 h, consistent with the manufacturer’s recommendations. Next, tissues were incubated at 37 °C overnight in a dry incubator. The development of blue color was then assessed.

Senescence-associated β-galactosidase (SA-β-gal) staining was used to identify senescent cells in renal tissue sections. Digital image analysis was performed on 10 representative fields for each condition and acquired at 40× magnification. SA-β-gal–positive nuclei were defined by the presence of a distinct blue reaction product and quantified following color-based thresholding with ImageJ software (ImageJ 1.54g). The density of positive nuclei was calculated by normalizing counts to the total tissue area and expressed as nuclei per mm^2^.

### 2.3. Indirect Immunofluorescence and Confocal Laser Scanning Microscopy

Paraffin-embedded formalin-fixed tissue sections from RCC tumor and peritumoral regions, as well as normal renal tissue, were processed for indirect immunofluorescence as previously described [24,35,60].

After deparaffinization and antigen retrieval, sections were incubated with the following primary antibodies: rat monoclonal IgG2a anti-PTX-3 (clone MNB4, Abcam, Cambridge, UK) diluted 1:100; rabbit monoclonal anti-IL-6 IgG (ab6672, Abcam) diluted 1:100; rabbit monoclonal IgG anti-p21 (ab109520, Abcam) diluted 1:500; mouse monoclonal IgG2a anti-p16 (SC-1661, Santa Cruz Biotechnology, Dallas, TX, USA) diluted 1:100. The following secondary antibodies were used: Alexa Fluor goat anti-rat IgG 488, Alexa Fluor goat anti-rabbit IgG 546, Alexa Fluor goat anti-mouse IgG2a 555 (all from Alexa, Thermo Fisher, Waltham, MA, USA). All secondary antibodies were diluted 1:250. To stain the nuclei, samples were incubated with TO-PRO diluted 1:3000 (Invitrogen-Molecular Probe, Thermo Fisher, Waltham, MA, USA). To ensure specificity of immunofluorescence signals, multiple validation steps were performed. Isotype-matched controls (rat IgG2a, rabbit IgG, mouse IgG2a) were applied at identical concentrations and under the same conditions as primary antibodies, and no detectable fluorescence was observed. Secondary-only controls (omitting primary antibodies) were included in each staining session and showed no background signal. Internal negative controls consisted of normal renal tissue, which physiologically lacks PTX3, IL-6, p21, and p16 expression. All staining procedures were repeated in at least three independent experiments to confirm reproducibility. After staining, the slides were then mounted in Gel Mount (Biomeda Corp., San Josè, CA, USA) and sealed.

Specific fluorescence was evaluated by confocal microscopy using the Leica TCS SP5 (Leica, Wetzlar, Germany) equipped with imaging Leica Application Suite software (LAS X ver. 5.3.1) and with argon-krypton (488 nm), green-neon (543 nm), and helium-neon (633 nm) lasers. Fluorescence quantification was performed as previously described [24,25,35]. In detail, to perform fluorescence quantification, up to 10 optical slices (0.6 μm thickness each) were acquired for every sample using a ×40 oil immersion differential interference contrast (DIC) objective. Image acquisition was carried out by an operator blinded to the origin of the slides, using identical photomultiplier tube settings, pinhole aperture, and laser voltage for all samples. Images were analyzed with the LAS X software (ver. 5.3.1). Each image consisted of 1028 × 1028 pixels covering an area of 387.5 × 387.5 μm^2^ and was recorded in line averaging mode (two scans) to minimize background noise. Emission spectra were collected by selecting specific wavelength domains: Alexa 488 was excited at 488 nm with an Argon/Krypton laser, and fluorescence emission was detected between 500 and 542 nm. Each 24-bit TIFF image was processed with LAS X software (ver. 5.3.1) to visualize the full z-series in x-y projection. Mean fluorescence intensity (MFI), defined as the sum of pixel intensities within the selected channel divided by the number of pixels in the region of interest, was quantified using dedicated LAS AF analysis tools.

### 2.4. IL-6 Serum Level Assessment

IL-6 serum levels were tested on serum samples drawn at the time of nephrectomy in the whole study population. Serum IL-6 levels in RCC patients were compared with those from 40 non-cancer volunteers, which served as the baseline reference group. Circulating IL-6 was quantitated using a commercially available ELISA Kit, according to the manufacturer’s instructions (R&D Systems, Minneapolis, MN, USA), as described elsewhere [61,62].

### 2.5. Statistical Analysis

Statistical analysis was performed using Statistical Package for Social Sciences (SPSS) 25.0 software (SPSS Inc., Evanston, IL, USA), as previously described [63,64,65,66]. The Shapiro–Wilk test was employed to assess the normality of variable distributions. For normally distributed variables, paired *t*-tests were applied, while comparisons of not normally distributed variables were evaluated using the Mann–Whitney *U* test. Categorical variables were compared using the chi-square (X^2^) test, as appropriate. For analyses involving multiple group comparisons (e.g., normal vs. peritumoral vs. tumor tissue), Bonferroni correction was applied to adjust *p*-values for multiple testing. A *p*-value < 0.05 after correction was considered statistically significant.

In cancer-specific survival (CSS) analyses, patients who died of RCC unrelated causes or were lost to follow-up were censored. Progression-free survival (PFS) was calculated from the date of surgery to the date of disease recurrence. Estimates of CSS and PFS were evaluated using the Kaplan–Meier method, and differences between groups were assessed with the log-rank (Mantel–Cox) test. To test the prognostic value of serum IL-6 (sIL-6), a bi-variable Cox proportional hazards model was conducted. Specifically, the prognostic analysis of sIL-6 for CSS and PFS were evaluated in separate models adjusted individually for Fuhrman grade and TNM stage, respectively. Hazard ratios (HRs) and 95% confidence intervals (CIs) were calculated.

Data were presented as mean ± standard deviation (SD) for normally distributed variables, median and interquartile range (IQR) for non-normally distributed variables, or percentage frequencies, unless otherwise specified. All analyses were conducted with two-tailed tests, and statistical significance was set at *p* < 0.05 unless otherwise specified.

## 3. Results

Clinical and pathological characteristics of the 57 patients with clear cell renal cell carcinoma (ccRCC) undergoing radical or partial nephrectomy, enrolled in the present study, are summarized in Table 1. The median age was 61 years, with 31.6% female patients. Fuhrman grading revealed a predominance of low-grade tumors (14.0% for G1 and 57.9% for G2, respectively), while TNM staging showed that most patients presenting with tumors localized to the kidney (pT2b or lower).

**Table 1:** Clinical and pathological characteristics of patients undergoing radical or partial nephrectomy for ccRCC and subjected to tissue analysis.

**Clinical Characteristics of RCC Patients**		
Patients, *n*		57
Age, median (range)		61 (25–87)
Female Gender, *n* (%)		18 (31.6%)
Diabetes Mellitus, *n* (%)		14 (24.6%)
high sensitive C reactive protein (hs-CRP); mg/dL		4.8 ± 1.2
CKD-EPI eGFR, mL/min/1.73 m^2^		89.5 ± 11.5
**Histologic Characterization of RCC Patients**		
Fuhrman grading	G 1	8 (14.0%)
	G 2	33 (57.9%)
	G 3	9 (15.8%)
	G 4	7 (12.3%)
TNM/AJCC Staging	pT1a	9 (15.8%)
	pT1b	24 (42.1%)
	pT2a	6 (10.5%)
	pT2b	5 (8.8%)
	pT3a	12 (21.1%)
	pT3b-c	1 (1.8%)
	pT4	0 (0.0%)
	pN+	8 (10.5%)
	cM+	1 (1.8%)

Abbreviations: CKD-EPI, Chronic Kidney Disease EPIdemiology Collaboration; eGFR, estimated Glomerular Filtration Rate; TNM/AJCC: Tumor size, Lymph Nodes affected, Metastases/American Joint Committee on Cancer. Values are expressed as median (range); mean ± standard deviation, or number of cases and (percentage).

**Figure 1:**
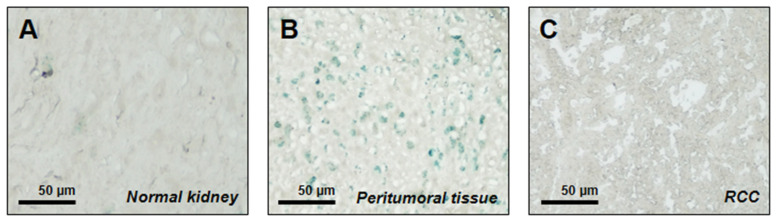
SA-β-gal staining of normal renal tissue (**A**), peritumoral tissue (**B**) and RCC (**C**). Representative images of 10 different patients were acquired by phase contrast microscopy (10× magnification). Bar = 50 µm.

To evaluate the presence of senescent cells in normal kidneys, as well as in both peri-tumoral and cancer tissues from RCC patients, SA-β-Gal staining was performed. Senescent cells were not identified within renal tissues from healthy control donors (Figure 1A), while surprisingly we observed higher enzymatic activity within apparently normal peri-tumoral tissues (Figure 1B) but not the RCC tumor core (Figure 1C). Quantitative analysis revealed a marked difference in SA-β-gal–positive cell density among the analyzed tissues. While peri-tumoral renal tissue exhibited a density of approximately 200 SA-β-gal–positive cells per mm^2^ (IQR 150–300), only few positive cells were detected in healthy renal tissue and in the tumor core, with densities of 20 (IQR 10–50) and 15 (IQR 5–90) cells per mm^2^, respectively (one-way ANOVA, *p* < 0.05).

These preliminary data led us to more carefully analyze the apparently normal peritumoral tissue in RCC specimens. Given the presence of senescent cells in peritumoral tissue, we next examined PTX3 because this long pentraxin is a key inflammatory mediator and a recognized SASP component. Its involvement in chronic inflammation and tissue remodeling makes PTX3 a biologically relevant marker to investigate in the context of peritumoral inflammaging [12,13,14,35].

**Figure 2:**
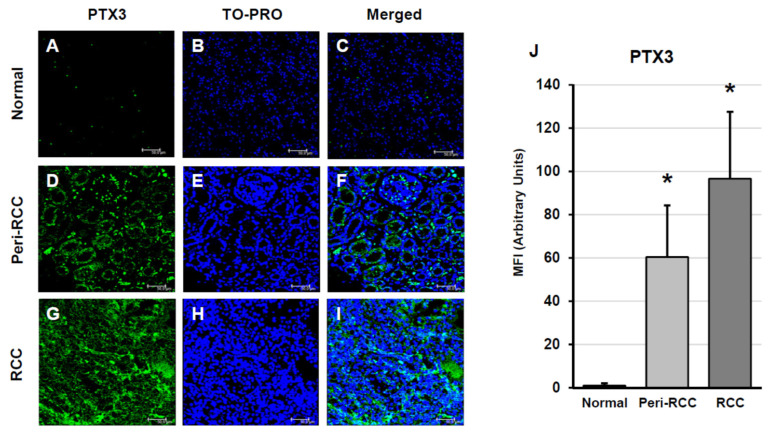
PTX3 expression in peritumoral and tumor regions of RCC tissues. The images presented are representative of data obtained in 3 independent experiments involving the study of 10 unrelated patients for each group: normal kidney (**A**–**C**), peritumoral tissue (**D**–**F**) and renal cancer tissue (**G**–**I**). The nuclei are stained with TO-PRO-3 (blue). (Scale Bar = 50 micron − 40× oil immersion objective − 400× magnification) (**J**) Fluorescence quantification analysis (* vs. normal kidney *p* < 0.001) is reported (histogram on the right).

An analysis of PTX3 expression by confocal microscopy showed that expression of this pentraxin was virtually absent in normal renal tissue (Figure 2A–C). However, there was a progressive and significantly increasing degree of PTX3 expression in peritumoral (pseudonormal) (Figure 2D–F) and intratumoral (Figure 2G–I) regions of the clinical specimens. Quantitative fluorescence analysis confirmed elevated PTX3 levels in both peritumoral and tumor tissues (*p* < 0.001 vs. control) (Figure 2J).

To explore whether the expression of PTX3 was related to a concurrent state of inflammation and aging, we evaluated patterns of expression of SASP (Senescence-Associated Secretory Phenotype) biomarkers, with IL-6, p21/CIP1/WAF1 and p16/INK4a explored.

**Figure 3:**
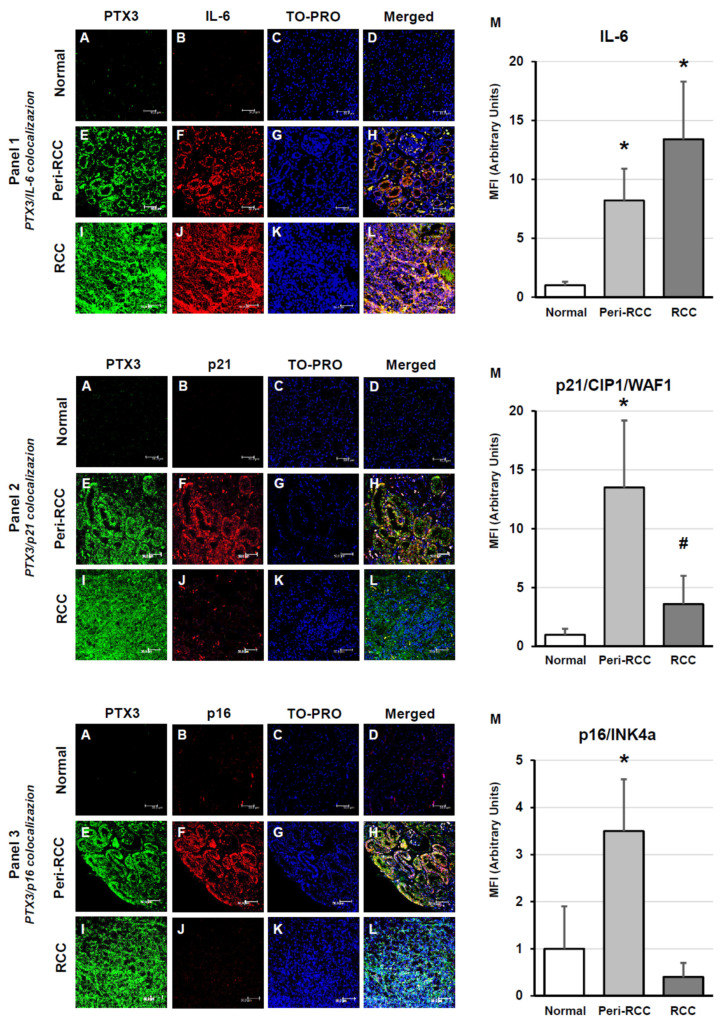
Expression of SASP-related factors in peritumoral and tumor regions of RCC tissues. Panel 1: PTX3/IL-6 colocalization; Panel 2: PTX3/p21 colocalization; Panel 3: PTX3/p16 colocalization. For each panel: (**A**–**D**): normal renal tissue; (**E**–**H**): peritumoral tissue; (**I**–**L**): renal cancer; (**M**): fluorescence quantification analysis. The images presented are representative of data obtained in 3 independent experiments involving study of 10 unrelated patients for each group. The nuclei are stained with TO-PRO-3 (blue). (Scale Bar = 50 micron − 40× oil immersion objective − 400× magnification) Fluorescence quantification analysis (* vs. normal kidney *p* < 0.001; # vs. normal kidney *p* < 0.01) is reported (right histograms in panels 1, 2 and 3).

IL-6 protein was expressed in a progressive manner in peritumoral and intratumoral RCC regions where it was strongly colocalized with PTX3 expression, while it was absent in normal kidney specimens (Figure 3, panel 1).

The cell cycle inhibitor p21/CIP1/WAF1 and the tumor suppressor p16/INK4a were absent in the normal tissues, while their expression was significantly increased within the apparently normal peritumoral tissue, where it colocalized with PTX3. However, expression ofp21 andp16 was suppressed or absent in the intratumoral areas, respectively (Figure 3, panel 2 and 3).

Quantitative fluorescence imaging analysis confirmed a strongly progressive increase of IL-6 expression in both peritumoral areas and intratumoral regions, while p21 and p16 were more highly expressed in peritumoral areas (*p* < 0.001) and significantly reduced (p21; *p* < 0.01) or absent (p16) in tumor regions (Figure 3, right histograms in panels 1, 2 and 3). The increased expression of p16 and p21 in peritumoral tissue indicates the presence of a senescent, growth-arrested microenvironment. In contrast, their marked reduction or absence in the tumor core suggests that RCC cells may bypass senescence-mediated cell-cycle arrest, supporting a shift toward a more proliferative and aggressive phenotype.

Based on morphological evaluation and spatial distribution, the expression of inflammaging-related markers in peritumoral renal tissue was predominantly localized to tubular epithelial structures. PTX3, IL-6, p16, and p21 immunoreactivity was mainly detected within tubular profiles adjacent to the tumor margin, while glomerular structures were largely negative. Senescence-associated β-galactosidase activity showed a similar distribution, being primarily confined to tubular compartments in peritumoral areas. A minor contribution from interstitial stromal cells and resident immune cells was also observed, particularly in close proximity to inflammatory infiltrates.

**Figure 4:**
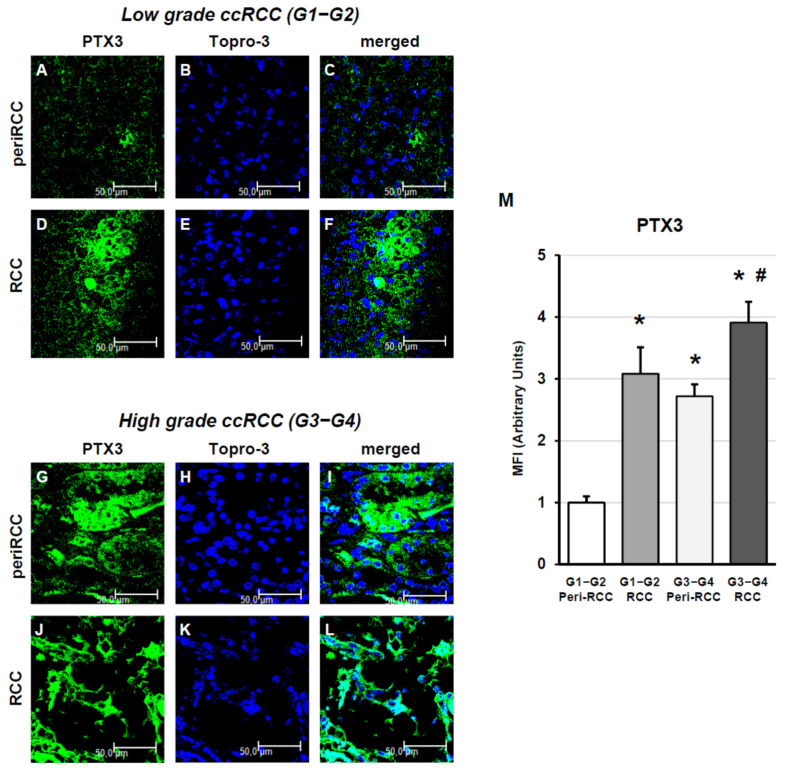
PTX3 expression according to grading in peritumoral and tumor regions of RCC tissues. (**A**–**C**): peritumoral tissue from low-grade RCC; (**D**–**F**): renal cancer tissue from low-grade RCC; (**G**–**I**): peritumoral tissue from high-grade RCC; (**J**–**L**): renal cancer tissue from high-grade RCC; (**M**): fluorescence quantification analysis. The images presented are representative of data obtained in 3 independent experiments involving study of 10 unrelated patients for each group. The nuclei are stained with TO-PRO-3 (blue). (Scale Bar = 50 micron − 40× oil immersion objective − 400× magnification). Fluorescence quantification analysis (* vs. G1–G2 peritumoral areas *p* < 0.001; # vs. G3–G4 peritumoral areas *p* < 0.01) is reported (right histograms).

We next analyzed PTX3 expression and SASP-related signals according to Fuhrman grade. In low grade ccRCC (G1–G2), PTX3 was weakly expressed in peritumoral area (Figure 4A–C) while it significantly increased in tumor regions (Figure 4D–F). In high grade ccRCC (G3–G4), the expression of PTX3 was elevated in apparently normal peritumoral areas (Figure 4G–I) and further increased in tumor regions (Figure 4J–L, fluorescence quantification analysis in Figure 4M).

**Figure 5:**
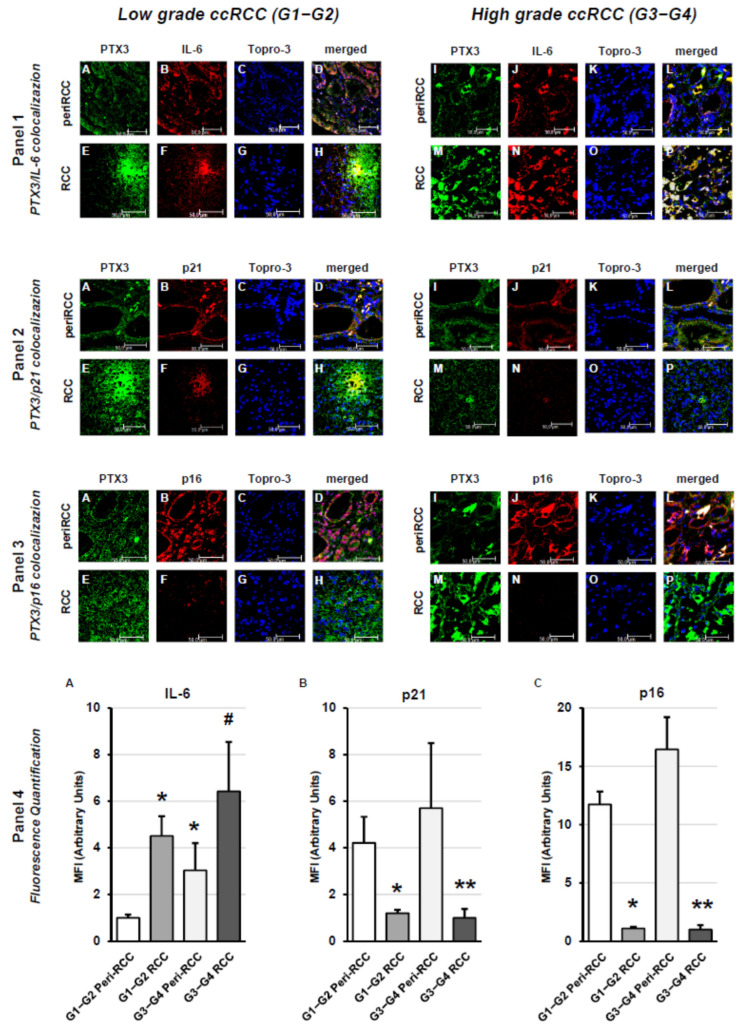
Tissue expression of SASP-related factors according to grading in peritumoral and tumor regions of RCC tissue. Panel 1: PTX3/IL-6 colocalization; Panel 2: PTX3/p21 colocalization; Panel 3: PTX3/p16 colocalization; Panel 4: fluorescence quantification analysis. For panels 1-2-3: (**A**–**D**): low-grade peritumoral tissue; (**E**–**H**): low-grade RCC; (**I**–**L**): high-grade peritumoral tissue; (**M**–**P**): high-grade RCC. For Panel 4: fluorescence quantification of IL-6 (**A**), p21 (**B**) and p16 (**C**) among different conditions. The images presented are representative of data obtained in 3 independent experiments involving study of 10 unrelated patients for each group. The nuclei are stained with TO-PRO-3 (blue). (Scale Bar = 50 micron − 40× oil immersion objective − 400× magnification). Fluorescence quantification analysis (* vs. G1–G2 peritumoral areas *p* < 0.001; # vs. G3–G4 peritumoral areas *p* < 0.01; ** vs. G3–G4 peritumoral areas *p* < 0.001) is reported (panel 4).

The analysis of SASP-related factors according to Fuhrman grade revealed that IL-6 was expressed in apparently normal peritumoral areas both in low grade ccRCC and, to significantly higher degree, in high grade ccRCC (Figure 5, panel 1), as confirmed by fluorescence quantitative analyses (Figure 5, panel 4, A). Moreover IL-6 expression significantly increased in intratumoral regions in both low grade ccRCC and high grade ccRCC (Figure 5, panel 1). The analysis of the cell cycle inhibitor p21/CIP1/WAF1 and the tumor suppressor p16/INK4a showed their significant expression in apparently normal peritumoral areas from both low grade ccRCC, with significantly higher expression observed for high grade ccRCC, while expression was significantly reduced (p21) or virtually absent in intratumoral regions in both low and high grade ccRCC (Figure 5, panels 2 and 3), as confirmed by fluorescence quantitative analysis (Figure 5, panel 4, B and C).

**Figure 6:**
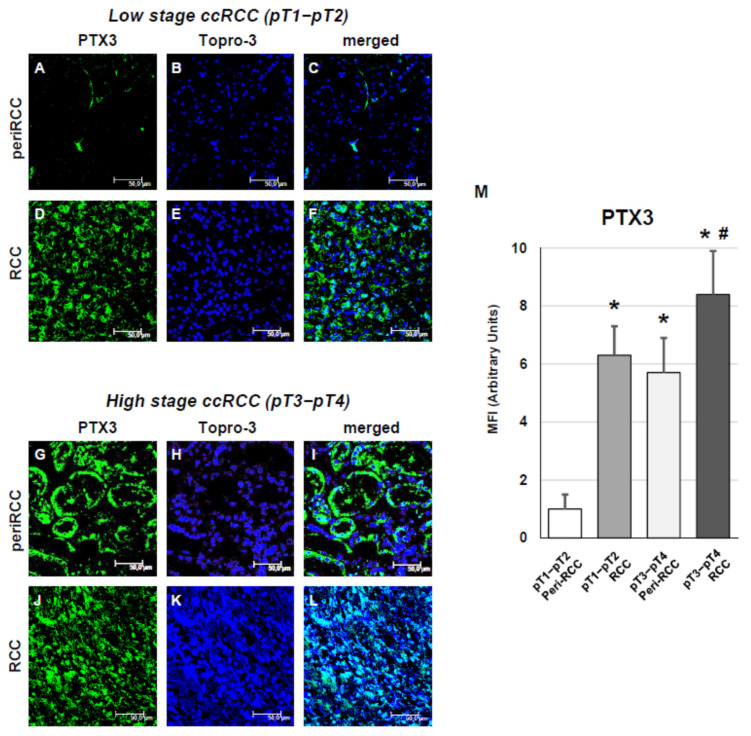
PTX3 expression according to TNM staging in peritumoral and tumor regions of RCC tissues. (**A**–**C**): peritumoral tissue from low-stage RCC; (**D**–**F**): renal cancer tissue from low-stage RCC; (**G**–**I**): peritumoral tissue from high-stage RCC; (**J**–**L**): renal cancer tissue from high-stage RCC; (**M**): fluorescence quantification analysis. The images presented are representative of data obtained in 3 independent experiments involving study of 10 unrelated patients for each group. The nuclei are stained with TO-PRO-3 (blue). (Scale Bar = 50 micron − 40× oil immersion objective − 400× magnification). Fluorescence quantification analysis (* vs. pT1–pT2 peritumoral areas *p* < 0.001; # vs. pT3–pT4 peritumoral areas *p* < 0.01) is reported (right histograms).

We next examined PTX3 expression and SASP-related markers according to TNM staging. In early-stage ccRCC (pT1–pT2), PTX3 showed weak expression in the peritumoral regions (Figure 6A–C), while its levels were markedly higher within tumor areas (Figure 6D–F). In advanced-stage ccRCC (pT3–pT4), PTX3 expression was already elevated in apparently normal peritumoral tissue (Figure 6G–I) and further increased in intratumoral regions (Figure 6J–L), as confirmed by fluorescence quantification (Figure 6M).

**Figure 7:**
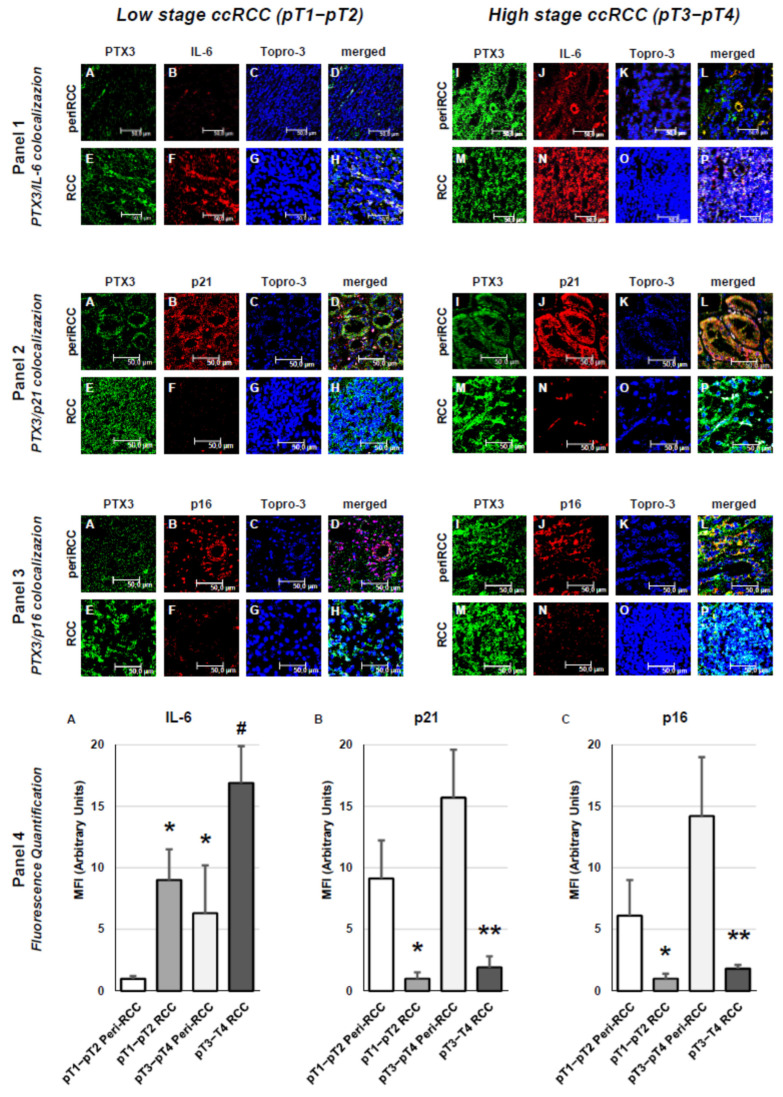
Tissue expression of SASP-related factors according to staging in peritumoral and tumor regions of RCC tissue. Panel 1: PTX3/IL-6 colocalization; Panel 2: PTX3/p21 colocalization; Panel 3: PTX3/p16 colocalization; Panel 4: fluorescence quantification analysis. For panels 1-2-3: (**A**–**D**): low-stage peritumoral tissue; (**E**–**H**): low-stage RCC; (**I**–**L**): high-stage peritumoral tissue; (**M**–**P**): high-stage RCC. For Panel 4: fluorescence quantification of IL-6 (**A**), p21 (**B**) and p16 (**C**) among different conditions. The images presented are representative of data obtained in 3 independent experiments involving study of 10 unrelated patients for each group. The nuclei are stained with TO-PRO-3 (blue). (Scale Bar = 50 micron − 40× oil immersion objective − 400× magnification). Fluorescence quantification analysis (* vs. G1–G2 peritumoral areas *p* < 0.001; # vs. G3–G4 peritumoral areas *p* < 0.01; ** vs. G3–G4 peritumoral areas *p* < 0.001) is reported (panel 4).

The evaluation of SASP-associated factors based on TNM staging revealed that IL-6 was detectable in peritumoral areas in both early-stage and, to a significantly greater extent, advanced-stage ccRCC (Figure 7, panel 1), as confirmed by fluorescence-based quantitative analysis (Figure 7, panel 4, A). Moreover, IL-6 expression was markedly higher in intratumoral regions across both stages (Figure 7, panel 1). Analysis of the cell cycle inhibitor p21/CIP1/WAF1 and the tumor suppressor p16/INK4a demonstrated substantial expression in peritumoral tissue from early-stage tumors, with significantly stronger expression in advanced-stage cases. Conversely, their expression was markedly reduced (p21) or nearly absent (p16) within tumor regions at both stages (Figure 7, panels 2 and 3), findings corroborated by fluorescence quantification (Figure 7, panel 4, B and C).

These expression patterns suggest that peritumoral tissue exhibits a senescent and inflammatory phenotype, whereas the tumor core progressively loses senescence-associated cell-cycle inhibitors. This transition is consistent with increased proliferative capacity and supports the notion that RCC cells may escape senescence-mediated growth arrest during disease progression.

**Figure 8:**
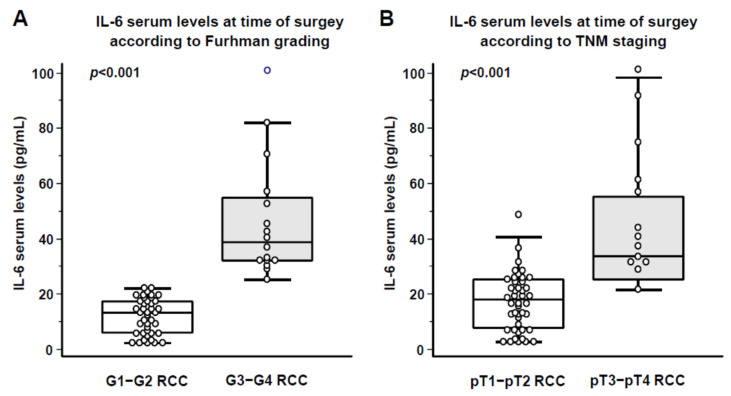
Serum levels of IL-6 at time of surgery in relation to Fuhrman grading (**A**) and TNM staging (**B**).

Compared to p21 and p16, IL-6 represents the SASP component most easily measurable in patient serum; therefore, subsequent analyses focused on IL-6. The marked tissue expression of SASP-related factors in apparently healthy peritumoral areas prompted us to assess serum IL-6 levels in patients enrolled in the study at the time of nephrectomy. We preliminarily compared serum IL-6 levels in the 57 RCC patients enrolled in the study with those measured in an age- and sex-matched control group of 40 healthy individuals. This analysis revealed that patients with RCC at the time of nephrectomy exhibited significantly higher circulating IL-6 concentrations compared to controls (21.8 ± 2.6 vs. 5.8 ± 0.6 pg/mL for RCC patients and healthy controls, respectively; *p* < 0.001). Subsequently, we compared serum IL-6 levels measured at the time of nephrectomy between patients with low versus high Fuhrman grade (Figure 8A) and between those with early versus advanced TNM stage (Figure 8B).

As shown in Figure 8A, patients with high-grade ccRCC (G3–G4) exhibited significantly higher serum IL-6 levels compared to those with low-grade ccRCC (G1–G2) (46.6 ± 21.4 vs. 12.2 ± 6.4 pg/mL, *p* < 0.001). Similarly, as reported in Figure 8B, patients with advanced-stage disease (pT3–pT4) showed markedly elevated IL-6 concentrations compared to early-stage cases (pT1–pT2) (49.5 ± 22.4 vs. 13.7 ± 8.6 pg/mL, *p* < 0.001).

**Figure 9:**
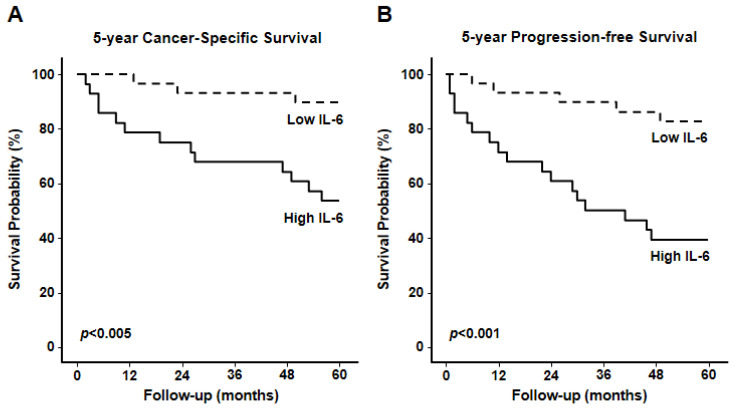
5-Year Cancer-Specific Survival (**A**) and Progression-Free Survival (**B**) in RCC patients according to serum IL-6 levels above or below the 50th percentile.

After stratifying the 57 RCC patients into two groups according to the 50th percentile of serum IL-6 levels (16.5 pg/mL), survival analyses were performed using Kaplan–Meier curves and the Log-rank Mantel–Cox test. Patients with IL-6 concentrations below the median exhibited a significantly higher 5-year cancer-specific survival compared to those with IL-6 above the median (Log-rank 89.7% vs. 53.6%, *p* < 0.005; Figure 9A). Similarly, Progression-free survival was significantly lower in patients with elevated IL-6, with 5-year PFS of 39.3% versus 82.8% in the low IL-6 group (Log-rank *p* < 0.001; Figure 9B). These findings underscore the strong prognostic impact of systemic IL-6 elevation on both cancer-specific and progression-free survival in RCC.

To assess the prognostic significance of serum IL-6 while accounting for the limited number of outcome events, bi-variable Cox regression models were applied. In these analyses, serum IL-6 was evaluated together with Fuhrman grade or TNM stage in separate models (Table 2). When adjusted for Fuhrman grade, elevated serum IL-6 levels remained significantly associated with reduced cancer-specific survival (CSS) and shorter progression-free survival (PFS) (HR 6.821, 95% CI 1.724–27.054, *p* = 0.006 for CSS; HR 4.378, 95% CI 1.516–12.6324, *p* = 0.007 for PFS, respectively). Similarly, serum IL-6 retained its prognostic significance in bi-variable models adjusted for TNM stage (HR 6.143, 95% CI 1.517–24.961, *p* = 0.0114 for CSS; HR 3.981, 95% CI 1.394–11.384, *p* = 0.010 for PFS, respectively).

Taken together, these findings indicate that systemic IL-6 levels provide prognostic information independent of tumor grade or stage when considered individually, supporting the role of IL-6 as a clinically relevant biomarker in clear cell renal cell carcinoma.

## 4. Discussion

This study provides novel insights for the role of inflammaging in the pathogenesis and progression of clear cell renal cell carcinoma (ccRCC). Traditionally, the peritumoral region has been considered histologically normal and often excluded from molecular analyses. However, our findings challenge this assumption by demonstrating that peritumoral tissues exhibit a distinct molecular profile characterized by increased expression of senescence-associated and inflammatory markers, including PTX3, IL-6, p21, and p16.

The concept of inflammaging—chronic, low-grade inflammation associated with aging—is viewed as increasingly relevant in the setting of cancer (immuno)biology [42,43,44,45,46,47]. In this context, the senescence-associated secretory phenotype (SASP) plays a pivotal role by promoting a pro-inflammatory milieu that influences neighboring cells and modulates the tumor microenvironment (TME) [67]. Our data show that PTX3 and IL-6 are significantly upregulated in both peritumoral and tumor regions of RCC tissues, with a progressive increase noted for normal to peritumoral to tumor regions in patient specimens.

Interestingly, while p21 and p16 were markedly expressed in peritumoral tissues, their levels of expression were significantly reduced or absent within the tumor core. The differential expression of p16 and p21 between peritumoral and intratumoral regions highlights a biologically relevant transition from a senescent phenotype in the peritumoral zone to a more proliferative, inflammation-driven progressive state within the tumor core. This pattern supports the hypothesis that RCC cells may escape senescence-induced growth arrest, a mechanism associated with tumor progression and poor prognosis [48,49,50,51]. This is particularly relevant in the case of high-grade tumors, where PTX3 and IL-6 expression was further elevated, and expression of senescence markers was suppressed. These findings are consistent with previous studies indicating that loss of senescence control is a hallmark of tumor progression and poor prognosis [52,53,54,55,56,57,68].

The progressive increase of PTX3 and IL-6 from peritumoral to intratumoral regions, together with the suppression of p21 and p16 in the tumor core, highlights a biologically relevant shift toward a more aggressive phenotype of RCC. This spatial gradient supports the hypothesis that inflammaging-related factors contribute to the transformation of the peritumoral niche into a pro-tumorigenic environment [24,25,35,44,59].

An additional relevant finding of this study is the cellular localization of inflammaging features within the peritumoral tissue. Based on morphological and spatial analysis, senescence- and inflammation-related markers were predominantly expressed by renal tubular epithelial cells rather than by tumor cells themselves. This observation is consistent with the recognized susceptibility of tubular epithelial cells to chronic stress, senescence, and SASP activation within inflammatory microenvironments. Although a minor contribution from stromal and immune cells cannot be excluded, the preferential tubular localization supports the concept that apparently normal renal parenchyma adjacent to the tumor acts as an active participant in shaping a pro-inflammatory and pro-tumorigenic niche.

The absence of senescence markers such as p16 and p21 within the tumor core is consistent with the molecular biology of clear cell RCC. Inactivation of the VHL gene, a hallmark of ccRCC, leads to constitutive stabilization of HIF-1α and HIF-2α, promoting transcriptional programs that favor proliferation, metabolic reprogramming, and angiogenesis. These pathways are known to suppress senescence checkpoints and facilitate escape from cell-cycle arrest [69,70]. Moreover, additional alterations frequently observed in ccRCC—including dysregulation of CDKN2A/B, p53 pathway alterations, and aberrant activation of mTOR signaling—further contribute to senescence bypass [71]. As a result, the tumor core becomes enriched in highly proliferative clones that have selectively eliminated or silenced senescence programs [72], whereas the peritumoral region retains a senescent phenotype driven by chronic stress and SASP signaling.

While our study provides spatial and molecular insights into the expression of inflammaging-related markers, we acknowledge the need for next-level functional validation. Future studies will investigate the mechanistic role of PTX3 and inflammaging in RCC progression, as well as their potential to serve as actionable therapeutic targets.

PTX3, a long pentraxin involved in innate immunity, has a dual role in cancer biology, acting as either a tumor suppressor or promoter depending on the context [16,17]. In RCC, PTX3 is produced locally by immune and epithelial cells in response to inflammatory stimuli [12,13,14,15,59], and its expression correlates with disease severity and patient prognosis [24,25,26,27,28,29,30,31,32,33,34,35]. Our findings support the pro-tumorigenic role of PTX3 in ccRCC, where it colocalizes with IL-6 and senescence markers within peritumoral tissues areas which suggests its involvement in the establishment of a SASP-like phenotype. Moreover, PTX3 has been implicated in modulating key oncogenic pathways including PI3K/AKT/mTOR and in the promotion of angiogenesis, epithelial-to-mesenchymal transition (EMT), and immune evasion [18,19,20,21,22,23].

IL-6, a central mediator of inflammation and SASP, was also significantly elevated in expression in both tissue and serum samples isolated from patients with high-grade and high-stage ccRCC. Elevated serum IL-6 levels were associated with significantly lower 5-year cancer-specific survival and progression-free survival, underscoring the potential utility of this cytokine as a prognostic biomarker [30,31]. Consistent with these tissue findings, serum IL-6 levels increased in tumors with a more aggressive clinical behavior, while patients above the median IL-6 (16.5 pg/mL) had markedly lower 5-year outcomes.

To assess the prognostic significance of serum IL-6 while accounting for the limited number of outcome events, we applied bi-variable Cox regression models rather than fully multivariable analyses. Within this framework, serum IL-6 retained a significant association with both cancer-specific and progression-free survival when adjusted separately for Fuhrman grade or TNM stage, supporting the additive prognostic value of systemic IL-6 beyond established clinicopathologic factors. These findings are biologically plausible, given the central role of IL-6 in the senescence-associated secretory phenotype (SASP), chronic inflammation, and tumor-promoting signaling cascades.

Although based on a small cohort, our findings are consistent with previous reports linking systemic inflammation to poor outcomes in RCC and other malignancies [10,11]. IL-6 may exert its effects through activation of the JAK/STAT3 signaling pathway, which promotes tumor cell survival, proliferation, and immune suppression [73,74].

From a clinical perspective, our findings suggest a clear association between elevated IL-6 levels (both tissue and circulating) and more aggressive disease. However, the observational nature of this study does not allow us to infer causality. IL-6 may act as a mediator of tumor-promoting inflammation, but it may also reflect systemic inflammatory responses, tumor burden, or paraneoplastic activity [75]. While IL-6 is mechanistically linked to STAT3 activation and RCC progression in experimental models [76], our data support its role as a prognostic biomarker rather than a proven driver of disease progression. These limitations have been acknowledged, and future mechanistic studies will be required to clarify the causal contribution of IL-6 to RCC biology.

From a therapeutic perspective, our findings also raise the clinically relevant question of whether targeting inflammaging-related pathways—particularly IL-6 signaling—could synergize with current therapeutic strategies for clear cell renal cell carcinoma. IL-6 acts as a central driver of the senescence-associated secretory phenotype and signals predominantly through the JAK/STAT3 axis, a pathway strongly implicated in RCC progression, immune evasion, and therapeutic resistance [77]. Experimental evidence indicates that IL-6 blockade reduces STAT3 activation and tumor cell viability in RCC models, supporting a biological rationale for targeting this pathway [78].

Although anti-IL-6 or anti-IL-6 receptor agents such as tocilizumab are not currently approved for oncologic indications in RCC, growing preclinical and emerging clinical evidence across solid tumors suggests that IL-6 inhibition may enhance antitumor immunity and improve responses to immune checkpoint inhibitors (ICIs). In particular, IL-6 blockade has been shown to modulate the inflammatory tumor microenvironment, reduce STAT3-driven immunosuppression, and potentially uncouple immune-related toxicity from immunotherapy efficacy [79].

In the context of RCC, where immune checkpoint blockade combined with tyrosine kinase inhibitors represents standard of care for advanced disease [80], modulation of IL-6–dependent inflammaging may represent a complementary strategy aimed at reshaping the peritumoral microenvironment. Our observation that peritumoral tissue exhibits a senescent, IL-6–rich phenotype—particularly in high-grade tumors—supports the hypothesis that targeting IL-6 signaling could be most relevant in biologically aggressive disease subsets.

Importantly, at present, these considerations remain hypothesis-generating. Prospective clinical trials are required to evaluate the safety, timing, and therapeutic benefit of combining IL-6 pathway inhibitors with established RCC treatments. Nonetheless, our data provide a biological framework supporting the inclusion of inflammaging-targeted strategies as adjuncts to existing immunotherapeutic and targeted approaches in ccRCC.

The differential expression of SASP-related markers according to Fuhrman grade and TNM stage further supports the relevance of inflammaging in RCC progression. In low-grade and low-stage tumors, PTX3 and IL-6 were only moderately expressed in peritumoral tissues and increased expression intensity within the tumor core. In contrast, high-grade and high-stage tumors exhibited strong expression of these markers even in the peritumoral zone, suggesting that the inflammatory and senescent phenotype is more pronounced and widespread in aggressive disease. This observation raises the possibility that early molecular changes in the peritumoral microenvironment may precede and facilitate tumor dedifferentiation and progression.

From a clinical perspective, these findings have relevant implications. First, they highlight the potential utility of PTX3 and IL-6 as biomarkers for disease severity and prognosis. Second, they suggest that the peritumoral tissue may serve as a cogent therapeutic target, particularly in high-grade or treatment-resistant RCC. Interventions aimed at modulating the SASP or blocking key inflammatory mediators such as IL-6 could potentially disrupt the pro-tumorigenic feedback loop, leading to improved patient outcomes. Third, the identification of a senescent and inflammatory phenotype in histologically normal tissue underscores the need to develop more refined diagnostic criteria that incorporate molecular profiling.

Despite these promising findings, several limitations of the present study should be acknowledged. This investigation was designed as a retrospective, single-center analysis and was based on a relatively limited cohort, which may restrict the generalizability of the results. However, the study included all consecutive patients for whom complete clinical follow-up and suitable tissue and serum samples were available, thereby providing a representative snapshot of real-world clinical practice.

The skewed distribution toward low-grade and low-stage tumors reflects the available caseload rather than a selection bias. Owing to the retrospective and exploratory nature of the study, and the absence of a predefined sample size calculation, a formal power analysis was not performed. Accordingly, the present findings should be regarded as hypothesis-generating.

Moreover, while the expression of key markers was assessed using robust immunofluorescence and ELISA-based approaches, functional studies will be required to elucidate the mechanistic pathways linking inflammaging-related signals to tumor progression. Future investigations should also address the cellular origin of SASP-associated factors and their crosstalk with immune and stromal components within the tumor microenvironment. Importantly, validation of these observations in larger, multicenter prospective cohorts will be essential to establish their clinical relevance.

## 5. Conclusions

In conclusion, this study highlights the critical role of inflammaging in clear cell renal cell carcinoma (ccRCC), particularly within the peritumoral microenvironment. Although histologically normal, peritumoral tissue exhibits a senescent and inflammatory phenotype, based on elevated expression of PTX3, IL-6, p21, and p16. These markers suggest a transition from senescence-associated growth arrest to inflammation-driven proliferation, especially in the case of high-grade tumors. The suppression of senescence markers in the tumor core and increased serum IL-6 levels in aggressive cases correlate with inferior cancer-specific survival, reinforcing IL-6 as a potential prognostic biomarker of poor prognosis. The spatial modulation of these factors supports the hypothesis that inflammaging actively contributes to RCC progression. These findings open new avenues for biomarker development and suggest that targeting inflammaging-related pathways may offer promising therapeutic strategies, particularly for high-grade or treatment-resistant RCC. Further research is needed to clarify the cellular sources and mechanisms driving this inflammatory-senescent axis in progressor RCC patients.

## Figures and Tables

**Table 2:** Bi-variable Cox regression analyses for Cancer-Specific Survival (A) and Progression-free Survival (B).

**(A) Cancer-Specific Survival**					
			**95% CI**		
**Variables**	**Adjustment Variable**	**HR**	**Lower**	**Higher**	***p*** **Value**
Serum IL-6 (≥ vs. <16.5 pg/dL)	Fuhrman Grade	6.821	1.724	27.054	0.006
Serum IL-6 (≥ vs. <16.5 pg/dL)	TNM stage	6.143	1.517	24.961	0.011
**(B) Progression-Free Survival**					
			**95% CI**		
**Variables**	**Adjustment Variable**	**HR**	**Lower**	**Higher**	***p*** **Value**
Serum IL-6 (≥ vs. <16.5 pg/dL)	Fuhrman Grade	4.378	1.516	12.6324	0.007
Serum IL-6 (≥ vs. <16.5 pg/dL)	TNM stage	3.981	1.394	11.384	0.010

CI: confidence interval; HR: hazard ratio.

## Data Availability

The datasets used and/or analyzed during the current study are available from the corresponding author upon reasonable request.

## Abbreviations

The following abbreviations are used in this manuscript:

AJCC	American Joint Committee on Cancer
CKD-EPI	Chronic Kidney Disease Epidemiology Collaboration
CRP	C-Reactive Protein
ELISA	Enzyme-Linked Immunosorbent Assay
EMT	Epithelial-to-Mesenchymal Transition
PTX3	Pentraxin 3
RCC	Renal Cell Carcinoma
SASP	Senescence-Associated Secretory Phenotype
TNM	Tumor, Node, Metastasis
